# Antimicrobial resistance (AMR) and plant-derived antimicrobials (PDA_m_s) as an alternative drug line to control infections

**DOI:** 10.1007/s13205-013-0180-y

**Published:** 2013-10-23

**Authors:** Jatin Srivastava, Harish Chandra, Anant R. Nautiyal, Swinder J. S. Kalra

**Affiliations:** 1 Department of Applied Sciences, Faculty of Environmental Science, Himalayan Institute of Technology and Management, BKT, NH 24, Lucknow, 227005 UP India; 2 Department of Medicinal and Aromatic Plants, School of Agriculture and Allied Sciences, High Altitude Plant Physiology Research Center, H.N.B. Garhwal University, Srinagar, Uttrakhand India; 3 Department of Chemistry, Dayanand Anglo Vedic College, Civil Lines, Kanpur, UP India

**Keywords:** Antimicrobial**-**resistant microbes, Efflux pumps, Antimicrobial resistance, Plant antimicrobial compounds

## Abstract

Infectious diseases caused by antimicrobial-resistant microbes (ARMs) and the treatment are the serious problems in the field of medical science today world over. The development of alternative drug line to treat such infectious diseases is urgently required. Researches on ARMs revealed the presence of membrane proteins responsible for effusing the antibiotics from the bacterial cells. Such proteins have successfully been treated by plant-derived antimicrobials (PDA_m_s) synergistically along with the commercially available antibiotics. Such synergistic action usually inhibits the efflux pump. The enhanced activity of plant-derived antimicrobials is being researched and is considered as the future treatment strategy to cure the incurable infections. The present paper reviews the advancement made in the researches on antimicrobial resistance along with the discovery and the development of more active PDA_m_s.

## Introduction

Increasing antimicrobial resistance (AMR) among microbes caused the emergence of new resistant phenotypes and further caused the development of new antimicrobial compounds (Goossens 2013). Infectious diseases caused by antimicrobial-resistant microbes (ARM) have been frequently reported since last few years (Vila and Pal 2010). About 440,000 new cases of multidrug-resistant tuberculosis (MDR-TB) are recorded annually, causing approximately 150,000 deaths all over the world. Recently, a joint meeting of medical societies, the first ever in India was held to tackle the challenges of antimicrobial resistance in developing world (Ghafur 2013). As a result of this conference “Chennai declaration” came into existence, initiating efforts through a national policy to control the rising trend of AMR in India and abroad (Ghafur et al. 2012).

Multidrug-resistant (MDR) microbes are resistant to three or more antibiotics (Styers et al. 2006), however; strains of *Mycobacteriumtuberculosis*, resistant to virtually all classes of antimicrobials have also been identified in the Kwa Zulu Natal Province of South Africa (Gandhi et al. 2006), a typical example of Extremely Drug-Resistant Tuberculosis (XDR TB) reported in 64 countries to date (World Health Organization 2011). The global emergence of MDRs is increasingly limiting the effectivity of the existing antibiotic drugs (Hancock 2005) for e.g. methicillin-resistant *Staphylococcus aureus* (MRSA) and vancomycin-resistant *Enterococci spp*. (Norrby et al. 2005). The development of resistance among the microbes is the result of continuous selection pressure of antibiotics and their surroundings causing genetic alterations (Bush 2004) which, are transferred to the next generation and reach out to the wider range of other geographical regions through the transfer of genetic information exchange between microbes (Amábile-Cuevas 2003) (Table 1 presents the examples of some of the common MDRs). In this review, attempt has been made to understand specific issues such as factors causing resistance, the role of developing world with a quick overview of plant-derived antimicrobials (PDA_m_) and synergistic compounds as an alternative drug line.

**Table 1:** Examples of plasmids carrying integron integrase carrying gene cassettes imparting resistance against antimicrobials

Plasmid gene cassette	Resistance against	Microbes (isolation)	Conjugative transfer	References
*pVN84*	MDR	*Vibrio* spp.	✓	Rajpara et al. (2009)
*MLS*_*B*_ [*erm*(*B*) & *erm*(*C*)]	Erythromycin	*Staphylococcus* spp.	✘	Schlegelova et al. (2008)
*grlA* or *gyr A*	Ciprofloxacin	*Staphylococcus* spp.	✘	Campion et al. (2004)
*pbp2X*	β-Lactam	*Staphylococcus* spp.	✘	Coffey et al. (1991)
*CTX*-*M*				
*aac*(*6*′)-Ib	Aminoglycoside	*Klebsiella pneumoniae*	✓	Soge et al. (2006)
*emr*(B)	Macrolide-lincosamide-streptogramin B	*K. pneumoniae*	✓	
*pla* TEM-1	Ampicillin	*K. pneumoniae*	✓	
*dfr*	Trimethoprim	*K. pneumoniae*	✓	
*p3iANG*				
*dfr*A15	Trimathoprim	*Vibrio cholerae*	✓	Ceccarelli et al. (2006)
*bla* PI	β-Lactam	*V. cholerae*	✓	
*qacH*	Quaternary ammonia-compounds	*V. cholerae*	✓	
*aadA8*	aminoglycosides	*V. cholerae*	✓	
*mecA*	Methicillin (MDR)	*S. aureus*	✘	Hiramatsu et al. (2002)
*qnr* (carried on class 1 integron)	Ciprofloxacin	*V. Cholerae*	✘	Fonseca et al. (2008)
*bla*_*MDL*-*1*_	Carbapenem	*Enterobacteriaceae*	✓	Kumarasamy et al. (2010)

## Factors causing AMR

Microbes comprise 50 % of total living biomass and are well-survived life forms on earth. There exists a sharp distinction between microbes as pathogenic and non-pathogenic although; one-way exchange of genetic elements (Amábile-Cuevas 2003) may confer the pathogenic characters to the non-pathogenic microbe. Pathogenic microbes cause infectious diseases in humans and animals and are treated with antibiotics. Antibiotics also known as antimicrobials are chemical substances, toxic for most of the life forms. Irrational and deliberate use of antibiotics, migration of infected individuals to other communities (Memish et al. 2003), prolonged use of medical health care systems in hospitals, hunger and malnutrition are some of the main causes of the development of resistance against antibiotics in the microbes (Byarugaba 2004; Vila and Pal 2010). Antimicrobial use in veterinary practices especially as food additives is one of the causes of development of AMRs in zoonotics that may spread to humans (Memish et al. 2003) through the food chain. In this connection, reports of Schlegelova et al. (2008) suggest, least chances of spreading of a resistant strain through the dairy products, however; improperly processed raw meat is strongly discouraged for human consumption in developed nations (Threlfall 2002).

### Molecular understanding of AMR

Microbes attain resistance very rapidly against most of the currently available antibiotics because of the adaptability feature conferred by plasmids. Table 1 presents the examples of such plasmids carrying integron and gene cassettes in most common MDRs which on transfer, widespread the resistance (Kumarasamy et al. 2010). Gram-negative (Kumarasamy et al. 2010) and Gram-positive bacteria (Grohman et al. 2003) both exhibit conjugative transfer of plasmids, a natural way of horizontal gene transfer for e.g. the horizontal transfer of plasmid in between *Vibrio fluvialis* and *Vibrio cholerae* conferring resistance to *V. fluvialis* (Rajpara et al. 2009). Recent cases of AMR development include *Pseudomonasaeruginosa* and *Acinetobacterbaumannii* resistant to nearly all antibiotics including the carbanems (Huang and Hsueh 2008). Antibiotic inactivation (degradation of antibiotics by the microbial enzymes e.g. transferase and β-lactamase) causes resistance in microbes (Wright 2005; Jacoby and Munoz-Price 2005), more than 1,000 such β-lactamases are identified till date (Bush and Fisher 2011). Different antibiotics have different mode of actions, therefore, their use is largely dependent on variety of traits other than resistance (Amábile-Cuevas 2010) which either undergo rapid enzymatic degradation or actively effused by the resistant bacteria. Efflux pump in MDRs was first described by Roberts (1996) for tetracycline and macrolide antibiotics. In general, efflux pumps act through membrane proteins of substrate specificity, effuse the antibiotics from the bacterial cell, resulting in a low intracellular ineffective concentration of the drug (Gibbons 2004; Thorrold et al. 2007) altering the permeability of membrane. In a study, staphylococcal accessory regulator (*sarA*) was reported to contribute promising role, imparting resistance in *S. aureus* (Riordan et al. 2006). In addition, Kuete et al. (2011) reported two efflux pumps viz., AcerAB-TolC (Enterobacteriaceae) and MexAB-OprM (*Pseudomonasaeruginosa*) imparting resistance in Gram-negative bacteria against natural products. AMR is a genetically-modified manifestation, linked to the point mutation in bacterial non-chromosomal DNA. As in case of MRSA, the resistance to methicillin is associated with acquisition of a mobile genetic element, *SCCmec*, which contains *mecA-*resistant gene (Okuma et al. 2002). Analytical procedure followed on *Escherichia coli* showed reversible function of class 1 integron integrase gene machinery under selective pressure (Díaz-Mejía et al. 2008). Similar results were also observed by Hsu et al. (2006) whereby *E*. *coli* MDR was found associated with the class 1 integron gene. Detailed mechanism of development of AMR among microbes has been extensively reviewed by Byarugaba (2010).

## Developing world: the factory of MDRs

**Fig. 1:**
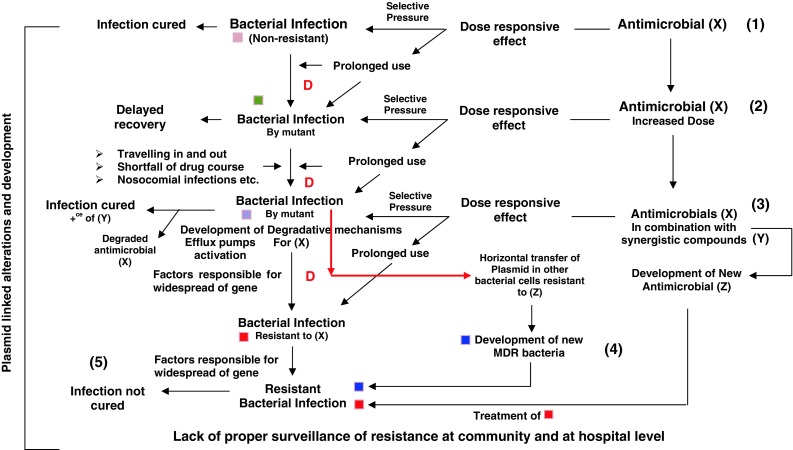
Illustrative sketch of the development of MDR microbes. The sketch is divided into various segments: (*1*) Bacterial infection was treated with calculated amount of antimicrobial drug (X) followed by complete cure, in the same time prolonged use of drug (X) put selective pressure causing point mutation (D). (*2*) Second infection (in a community only) was treated with same drug (X) with a higher dose, a delayed response was displayed because of mutant bacterial strain, (*3*) Third time infection (in a community only) trigger the resistance, in particular microbe for a particular drug (X); therefore, synergistic compounds (Y) were administered along with (X) may be for clinical trials, the successful treatment, leading to the production of new antimicrobial drug (Z), (*4*) Since the earlier bacteria attained resistance in due course of time for the drug (X) transferred the resistant gene into another strain of same species of bacteria resistant to the drug (Z) which was introduced in this community from the other one, gene cassettes got recombined on the plasmid to confer multi-drug resistant status to the new introduced bacteria. Infection caused by both these bacteria might be having same symptoms which would be treated with the newly developed drug (Z) keeping the resistance against (X) in consideration. (*5*) Infection could not be cured because the drug was applied to cure the (X) drug-resistant bacteria however; another bacteria having resistance against (Z) remained as such

Developing world especially the countries of South East Asia, Western and Central Africa, India and Pakistan are the most vulnerable for various infectious pandemic diseases. Byarugaba (2004) comprehensively reviewed and reported the AMR in developing countries. Several factors are associated with the AMR development including nosocomial infections, unsafe disposal of biomedical waste, inappropriately used antibiotics, self drug abuse, shortfall of antibiotic course and lack of mass awareness of infectious diseases and personal hygiene (Okeke et al. 2005a, b). In addition to these, lack of surveillance data, providing information of microbial infections common to a geographic location and the invasive microbial species have been suggested as the major causes of MDRs development in developing countries (Okeke et al. 2005a, b; Giske and Cornaglia 2010; Kartikeyan et al. 2010; Lalitha et al. 2013). Giske and Cornaglia (2010) emphasized on the surveillance practices especially the monitoring and sampling techniques of invasive microbial isolates. Surveillance of resistance in many developing countries is suboptimal (Okeke et al. 2005b) and unable to present the real picture of infectious diseases and the medication. Recent reports of Lalitha et al. (2013) showed the feasibility of proper surveillance of resistance by carrying experimental surveillance study on the school children in different geographic locations of Indian subcontinent. In India for *Salmonellatyphi*, MDR has become a norm in strains. This widespread resistant bacterium is associated with contaminated water supply in developing countries and through food products such as contaminated meat in developed countries (Threlfall 2002). Remarkable report of Kumarasamy et al. (2010) provides sufficient evidences in support of the positive role of developing world in the development of ARMs. Resistance to carbapenem conferred by plasmid encoded New Delhi metallo-β-lactamase-1 (*bla*_*NDM*-1_) is a worldwide health problem, especially in UK, (Kumarasamy et al. 2010) having the roots in India and Pakistan. The selective pressure on the bacterial cells is associated with the adaptations causing resistance among microbes for multiple antimicrobials for e.g. genes encoding NDM-1, OXA-23 and OXA-51 enzymes (hydrolyzing specific antibiotics) were observed in three different isolates of *Acinetobacter baumannii* in India (Kartikeyan et al. 2010). Alterations in gene structure were reported in *A. baumannii* as a result of selection pressure of antibiotics (Kartikeyan et al. 2010). The literature suggest, substandard surveillance of resistance, non-prescribed antibiotic usage causes huge selection pressure resulting in the development of AMR in developing countries and their suburbs (Byarugaba 2004; Okeke et al. 2005b; Kumarasamy et al. 2010). Figure 1 shows a schematic diagram showing the development of MDR microbe in community.

## Plants derived antimicrobial (PDA_m_): a ray of hope

 Antimicrobial resistance is rapidly increasing along with the development of classical antibiotics consequently, there is an urgent need to develop a different drug line to treat and control MDR bacterial infections. Medicinal values of plants were known to earlier traditional medical practitioners (Emeka et al. 2012). PDA_m_ substances are plant-originated secondary metabolites and have great concern because of their antibiotic activity without conferring resistance (Baris et al. 2006; Palaniappan and Holley 2010). PDA_m_s are classified as antimicrobial on the basis of dose ranging from 100 to 1,000 μg ml^−1^ for the minimum inhibitory concentration (MIC) susceptibility test performed on bacteria (Tegos et al. 2002). Table 2 presents few of the examples of plants and their active antimicrobial compounds. Plants have unlimited ability to produce wide variety of secondary metabolites most of which are aromatic compounds including alkaloids, glycosides, terpenoids, saponins, steroids, flavonoids, tannins, quinones and coumarins (Das et al. 2010) forming the basis of PDA_m_ compounds (Table 3). Target specific plant’s secondary metabolites having potential to treat and control the infections are being screened out globally for e.g. Coumarins having specificity on *Staphylococcus aureus* and ineffective on Gram-negative bacteria (Lewis and Ausubel 2006). The literature such as Cowan (1999); Lewis and Ausubel (2006) and González-Lomothe et al. (2009) provides comprehensive information on the major secondary metabolites of plant origin. Precise mechanistic approach of PDA_m_ and their activity on microbes has been discussed by Lewis and Ausubel (2006). In general, PDA_m_s (mostly secondary metabolites) are phenol derivatives, sufficiently able to control microbes by reducing pH, increasing membrane permeability, altering efflux pumping. Examples mentioned in Table 2 followed by recent studies of (Machado et al. 2003; Ram et al. 2004; McGaw et al. 2008; Renisheya et al. 2011; Ahmed et al. 2012; Emeka et al. 2012; Upadhyaya 2013) and the references there in, suggest the antimicrobial potential of various local and exotic plant species, although very few reports have suggested the mechanism of their actions. The affectivity of PDA_m_s largely depends upon the extraction methods (Das et al. 2010). In a study carried out by our group, methanolic, ethanolic and water extracts of several plants species viz., *Argemone maxicana*, *Callistomon lanceolatus*, *Allium sativum*, *Swietenia mahogany*, *Citrulus colocynthis*, *Salvadora persica*, *Madhuca Indica*, *Acacia nilotica* and *Pongamia pinnata* were assayed for their antimicrobial activity on most of the common MDRs viz., *Staphylococcus aureus*, *Bacillus cereus*, *B. pumilus*, *Klebsiella pneumonia*, *Salmonella typhi*, *E. coli* exhibiting activity of all the extracts, however; the target specificity of plant extracts could not be established because of uncertain mechanism of plant-derived antimicrobial compounds. A generalized mechanism of PDA_m_s on microbes suggests the effects of efflux pumping on MDRs: increasing permeability and reduce selection pressure (Lewis and Ausubel 2006). Antimicrobial peptides (AMPs) are also produced by plants against the infections also called as defensins. Plant defensins are small basic peptides, having characteristic 3D folding pattern, stabilized by eight disulfide linked cysteines (Thomma et al. 2002). AMPs have antimicrobial properties too (Li et al. 2012) and have been suggested as an alternative approach to improve treatment outcome (Brouwer et al. 2011), for e.g. IbAMP1, a plant originated disulfide linked β-sheet antimicrobial peptide (Wang et al. 2009).

**Table 2:** Plant derivatives as antimicrobial for the treatment of microbial infections

Plants	Plant derivatives	Effective against	References
*Medicago sativa*	Saponins, canavanine	*Enterococcus faecium Staphylococcus aureus*	Aliahmadi et al. (2012)
*Onobrychis sativa*	AMPs (antimicrobial peptides)	*E. faecium*, *S. aureus*	Aliahmadi et al. (2012)
*Allium sativum*	Organosulfur compounds (phenolic compounds)	*Campylobacter jejuni*	Lu et al. (2011)
*Raphanus sativum*	RsAFP2 (Antifungal peptide)	*Candida albicans*	Aerts et al. (2009)
*Vetiveria zizanioides* L. Nash	Vetivone (vetiver oil)	*Enterobacter* spp.	Srivastava et al. (2007)
*Chelidonium majus*	Glycoprotein	*B. cereus*, *Staphylococcus* spp.	Janovska et al. (2003)
*Sanguisorba officinalis*	Alkaloids, antimicrobial peptides	*Ps. aeruginosa*, *E. coli*	Janovska et al. (2003)
*Cinnamomum osmophloeum*	Cinnamaldehyde (in essential oil)	*Legionella pneumophila*	Chang et al. (2008)
*Ocimum basilicum*	Essential oil	*Salmonella typhi*	Wan et al. (1998)
*Micromeria nervosa*	Ethanolic extract	*Proteus vulgaris*	Ali-Shtayeh et al. (1997)
*Rabdosia trichocarpa*	Trichorabdal A	*Helicobacter pylori*	Kadota et al. (1997)
*Melaleuca alternifolia* and *Eucalyptus* sp.	Essential oil	*Staphylococcus* spp. and *Streptococcus* spp.	Warnke et al. (2009)
*Anthrocephalous cadamba* and *Pterocarpus santalinus*	Ethanolic extract	MDRs^M^	Dubey et al. (2012)
*Lantana camara* L.	Leaf extract in dichloromethane & methanol	MDRsG + ve and MDRsG−ve	Dubey and Padhy (2013)
*Butea monosperma* Lam.	Ethanolic and hot water extract of leaf	MDRs^M^	Sahu and Padhy (2013)
*Jatropha curcas* (Linn.)	Ethanolic and methanolic extract	MDRsG + ve + *Micrococcus* sp. & MDRsG−ve + *Shigella* sp. + *Bacillus* sp.	Igbinosa et al. (2009)
*Ficus exasperate* and *Nauclea latifolia*	Methanolic extract of leaf and stem	*E. coli*, *Shigella dysenteriae*, *S. typhi*, *C. albicans*, *P. aeruginosa*	Tekwu et al. (2012)
*Rhus coriaria*	Ethanolic extract	MDR *P. aeruginosa*	Adwan et al. (2010)

MDRsM = *Staphylococcus aureus* + *Acinetobacter* sp. + *Citrobacter freundii* + *Chromobacterium violaceum* + *Escherichia coli* + *Klebsiella* sp. + *Proteus* sp. + *Pseudomonas aeruginosa* + *Salmonella typhi* + *Vibrio cholera*; MDRsG + ve = *S. aureus* (MRSA) + *Streptococcus pyogenes* + *Enterococcus faecalis* (VRE); MDRsG−ve = *Acinetobacter baumannii* + *Citrobacter freundii* + *Proteus mirabilis* + *Proteus vulgaris* + *Pseudomonas aeruginosa*

**Table 3:** Examples of plant derivatives and their antimicrobial activities

Plant-derived antimicrobial groups	Structure	Chemical properties	Effective on microbes	References
Quinones		Conjugated cyclic-dione structure with molecular formula C_6_H_4_O_2_ e.g. Anthraquinone from *Cassia italica*	*Pseudomonas pseudomallei*, *Bacillus anthracis*, *Corynebacterium pseudodiphthericum*, *Pseudomonas aeruginosa*	Kazmi et al. (1994)
		6-(4,7 Dihydroxy-heptyl)quinone	*Staphylococcus aureus*, *Bacillus subtilis*, *Proteus vulgaris*	Ignacimuthu et al. (2009)
Alkaloids		Naturally occurring amines having nitrogen in heterocyclic ring of compounds and are the derivative amino acids e.g. glabradine from tubers of *Stephania glabra*	*S. aureus*, *S. mutans*, *Microsporum gypseum*, *M. canis*, *Trichophyton rubrum*	Semwal and Rawat (2009)
		l-Proline derived Monophyllidin from *Zanthoxylum monophyllum*	*Enterococcus faecalis*	Patino and Cuca (2011)
Lectins and polypeptides	–	Lectins are carbohydrate binding proteins (phytoaglutinin) with MW around 17,000–400,000	*E. coli*, *P. aeruginosa*, *Enterococcus hirae*, *Candida albicans* (fungi)	(Zhang and Lewis (1997)
Flavones/flavonoids/flavonols		Are ubiquitous in plant’s parts, fruits, seeds, flowers and even honey. Flavones are hydroxylated phenolics containing one carbonyl group	MDR *Klebsiella pneumoniae*, *P. aeruginosa*, *E. coli*	Özçelik et al. (2008); Edziri et al. (2012)
Coumarins		Coumarins are phenolic substances made of fused benzene and alpha pyrone ring forming toxic compounds found in plants such as *Dipteryx odorata*, *Anthoxanthum odoratum* etc	*S. mutans*, *S. viridans*, *S. aureus*	Widelski et al. (2009); Lewis and Ausubel (2006)
Terpenoids and essential oils		Isoprene derivatives having a general formula C_10_H_16_ therefore also called as Isoprenoids. Well-known examples include menthol	*S. viridans*, *S. aureus*, *E. coli*, *B. subtilis*, *Shigella sonnei* (highly active) *P. aeruginosa*, *E. coli*, *S. aureus*, *T. mentagrophytes* (low activity)	Banso (2009); Ragasa et al. (2008)
Tannins		Large polyphenolic compound containing sufficient hydroxyls and other suitable groups	*S. aureus*, *S. typhimurium*,	Moneim et al. (2007)

Chemical structure given in front of corresponding group of antimicrobials is not to be considered as generalized one, the references are in correspondence with bacteria

## Synergistic actions of PDA_m_s

The AMR is conferred by several factors which have already been reviewed in previous sections. Plasmid encoded resistance facilitate bacterial cells to develop resistance of various degrees. For instance, unlike Gram-positive, MDR Gram-negative bacterial species have developed a sophisticated permeability barrier as outer membrane comprised of hydrophilic lipopolysaccharide restricting the entry of hydrophobic (quinones and alkaloids) and amphipathic antibiotic compounds (Lewis and Ausubel 2006). The biased effect of PDA_m_s on Gram-positive and -negative species has been a key to the discovery of the synergistic compounds of plant origin (Lewis 2001). Plant antimicrobials act well in combinations with other amphipathic compounds. In addition to this, resistance in MDRs conferred by efflux pumping can be treated with the synergistic combinations of antimicrobial with an efflux pump inhibitor (EPI) and altering outer membrane permeability of MDR bacteria providing an effective drug (Savage 2001; Gibbons 2004; Baskaran et al. 2009). Studies of Chusri et al. (2009) reported another example of synergistic effect of plant-derived phenolics such as Ellagic acid (a derivative of Gallic acid) a non-antimicrobial, administered as EPI in combination with classical antibiotic to control *Acinetobacter baumannii.* Another example belongs to the well-studied plant *Berberis fremontii* and its amphipathic cation berberine inhibits the NorA MDR pump of *Staphylococcusaureus* when applied in combination with 5′-MHC (5′-methoxyhydnocarpin, an amphipathic weak acid) a real inhibitor of the pump enhancing the activity of berberine (Stermitz et al. 2000). Similar non-antimicrobial compounds known to enhance effectivity of antimicrobials have been discussed by Lewis (2001). Detailed mechanism of PDA_m_s on MDR *S. aureus* has been discussed in the review by Gibbons (2004). Wang et al. (2009) defined that the role of AMP plant defensin IbAMP1 isolated from plant *Impatiens balsamina* have a prime target, intercellular components, forming small channels that permit the transit of ions or protons across the bacterial membrane, the same activity was also observed in the linear analogs of this peptide.

## Future studies

Researches on the AMR and alternating drug system are endless and a lot of scope is there in the field of ethno-pharmacology. Scientists are working on the development of safe and effective antimicrobials all over the world. Future studies may involve the development of new plant-derived synergistic compounds capable of enhancing the activity of PDA_m_s. A lot of research potential is also there to answer the questions for e.g. mechanism of resistance in different bacterial species, development of XDRs and their control.

## Conclusion

AMR is a worldwide problem. Research literatures suggest that the substandard living in major parts of developing world is one of the major causes of the development of resistance among bacteria. The developed world is also vulnerable of getting widespread infections for e.g. USA is surrounded by the developing countries having high rates of resistance development. Nosocomial, water borne, health care systems and food products especially meats are some of the most common means of widespread of resistant gene globally. Thanks to the modern molecular approaches for making better understanding of the pathways of resistance development and its remedy. Pharmacologists are developing new antibiotic drugs to treat and control various infections, however; the chances of the development of resistance are equal to the emergence of new drugs. In addition, research suggest that the combinations of PDA_m_s and the synergistic compounds work efficiently on resistant strains ensuring no further resistance development. Moreover; concerted efforts have been solicited by the world community because poor countries are worst affected by the antimicrobial resistance and the developed countries are no longer safe (Diáz-Granados et al. 2008). In this regard, PDA_m_s in combination with plant-derived synergistic compounds may be the cost-effective approach to deal with global antimicrobial resistance.

## Abbreviations

MDR: Multidrug-resistant
XDR: Extensively drug-resistant
AMR: Antimicrobial resistance
PDA_m_: Plant-derived antimicrobials
ARM: Antimicrobial-resistant microbes
EPI: Efflux pump inhibitor
AMP: Antimicrobial peptides
